# Imaging Predictive Factors of Abdominal Aortic Aneurysm Growth

**DOI:** 10.3390/jcm10091917

**Published:** 2021-04-28

**Authors:** Petroula Nana, Konstantinos Spanos, Konstantinos Dakis, Alexandros Brodis, George Kouvelos

**Affiliations:** 1 Vascular Surgery Department, Larissa University Hospital, Faculty of Medicine, School of Health Sciences, University of Thessaly, 41110 Larissa, Greece; spanos.kon@gmail.com (K.S.); kostasdakis1994@gmail.com (K.D.); geokouv@gmail.com (G.K.); 2 Neurosurgery Department, Larissa University Hospital, Faculty of Medicine, School of Health Sciences, University of Thessaly, 41110 Larissa, Greece; alexgbrodis@yahoo.com

**Keywords:** imaging, abdominal aortic aneurysm, prediction, growth

## Abstract

Background: Variable imaging methods may add important information about abdominal aortic aneurysm (AAA) progression. The aim of this study is to assess available literature data regarding the predictive imaging factors of AAA growth. Methods: This systematic review was conducted using the PRISMA guidelines. A review of the literature was conducted, using PubMed, EMBASE and CENTRAL databases. The quality of the studies was assessed using the Newcastle-Ottawa Scale. Primary outcomes were defined as AAA growth rate and factors associated to sac expansion. Results: The analysis included 23 studies. All patients (2244; mean age; 69.8 years, males; 85%) underwent imaging with different modalities; the initial evaluation was followed by one or more studies to assess aortic expansion. AAA initial diameter was reported in 13 studies (range 19.9–50.9 mm). Mean follow-up was 34.5 months. AAA diameter at the end was ranging between 20.3 and 55 mm. The initial diameter and intraluminal thrombus were characterized as prognostic factors associated to aneurysm expansion. A negative association between atherosclerosis and AAA expansion was documented. Conclusions: Aneurysm diameter is the most studied factor to be associated with expansion and the main indication for intervention. Appropriate diagnostic modalities may account for different anatomical characteristics and identify aneurysms with rapid growth and higher rupture risk. Future perspectives, including computed mathematical models that will assess wall stress and elasticity and further flow characteristics, may offer valuable alternatives in AAA growth prediction.

## 1. Introduction

Abdominal aortic aneurysm (AAA) is a progressive disease, associated with an increase of the sac diameter during time [1,2]. Currently, AAA diameter remains the most applied and significant marker of growth [2]. Smaller aneurysms (<50 mm) present a slower rate of growth (estimated at 1.3 mm/year), while larger aneurysms increase up to 3-fold more [1]. Individualized factors, such as smoking or diabetes, have been proved to alternate AAA evolution positively or negatively, while sex does not seem to affect AAA growth rate [3].

Many clinical trials have focused on different medical factors, which could affect the limitation of growth of small AAA by targeting some of the pathways that seem to be associated with AAA formation and growth [4,5,6,7,8,9,10]. Pharmaceutical factors as angiotensin-converting enzyme inhibitors, beta-blockers, statins, metformin and antibiotics that could affect or reverse AAA expansion, are under evaluation [4,11]. Despite the undergoing research, current recommendations suggest AAA surgical or endovascular repair when its diameter exceeds 55 mm [1,12]. Thus, standardized reproducible imaging methods and newer imaging assessments are mandatory and may add important information about AAA progression and further, management.

The aim of this study is to assess available literature data regarding the predictive imaging factors of AAA growth.

## 2. Methods

### 2.1. Eligibility Criteria

For the methodology of this systematic review, analysis and inclusion criteria for study enrollment were pre-specified. The current systematic review was based on the Preferred Reporting Items for Systematic Reviews and Meta-Analysis statement (PRISMA) [13]. Two independent reviewers (P.N., K.S.) proceeded with data extraction using a non-blinded standardized method. Discrepancies were resolved by a third reviewer (G.K.). As the current analysis is a systematic review, no informed consent was required. Only articles in English were included. The main criterion was that all the included studies reporting on imaging findings, irrespectively of the method, associated with AAA growth in patients without previous open or endovascular repair.

### 2.2. Search Strategy

A search of the medical literature was conducted, using PubMed, EMBASE and CENTRAL databases, until September 30, 2020. The P.I.C.O. (patient/population; intervention; comparison; outcomes) model was applied to precisely implement the clinical questions and article selection, as presented in Table 1 [14]. Expanded Medical Subject Headings (MeSH) were used in multiple combinations: “imaging”, “abdominal aortic aneurysm”, “prediction” and “growth”. The primary selection was made according to the title and abstract while a secondary process was accomplished according to full texts.

### 2.3. Data extraction and Quality Assessment

A standardized Microsoft Excel file was conducted for data extraction. Extracted data included name of author, journal, date of publication, type of study (prospective or retrospective) and study period. Additionally, baseline demographics (age, sex), type of imaging modality (computed tomography (CT), magnetic resonance imaging (MRI), positron emission tomography (PET), ultrasonography (US)), initial AAA diameter, AAA diameter at follow-up, growth rate and other anatomical features, such as discontinuity of the wall, peak wall stress, wall thickness, AAA area and volume, type of thrombus and calcification, were collected.

The quality of observational studies was assessed using the Newcastle-Ottawa Scale (NOS) for cohort studies (Supplementary Table S1). This tool evaluates three main methodological domains of cohort studies—a. selection methods (representativeness of the exposed cohort, selection of the non-exposed cohort, ascertainment of exposure and demonstration that outcome of interest was not present at the start of the study); b. comparability of cohorts on the basis of the design or analysis; and c. assessment of outcomes (ascertainment of outcome, adequacy of follow-up). The scale uses a star system with a maximum of nine stars. Studies achieving at least six stars were considered to be of higher quality [15].

### 2.4. Outcomes

Primary outcomes were defined as the abdominal aneurysm growth rate in patients that had no previous repair and factors associated with aneurysm sac expansion.

### 2.5. Statistical Analysis

Only descriptive data are presented in the current review.

## 3. Results

**Figure 1:**
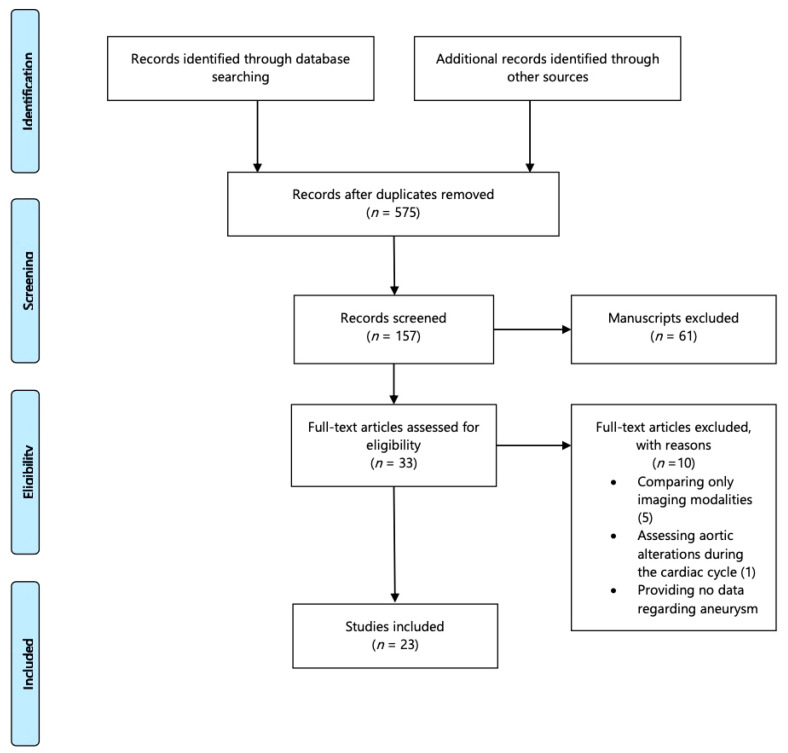
The flow chart of the selection process according to Systematic Reviews and Meta-Analysis statement [13].

Initially, 621 articles potentially suitable for inclusion were collected. After title and abstract exclusion due to no relevance to the topic, 33 full texts were assessed for eligibility. Twenty-three articles (published between 1994–2020) with 2244 patients were finally included, as depicted in Figure 1. Only observational cohort studies were included (10 prospective and 13 retrospective) with study cohorts ranging between 5 and 414 patients [16,17,18,19,20,21,22,23,24,25,26,27,28,29,30,31,32,33,34,35,36,37,38]. All patients suffered from AAA (mean age; 69.8 years (range 59.0–78.4 years), males; 1.545/1.815, 85%) and underwent imaging with different modalities. The initial evaluation was followed by one or more studies to assess AAA expansion. Different modalities were applied including CTA, MRI, PET and US or a combination of them. Furthermore, computational finite elements were used in seven studies to assess hemodynamic characteristics that would address aneurysm expansion (Table 2). CTA was the most applied method used to estimate sac diameter [16,18,19,23,24,25,26,29,30,36,37], while different combinations of CTA and other modalities have been used; angiography [17], PET-CT and MRI [27,38]. The CTA was used as the baseline imaging in modern mathematical models to estimate sac expansion [23,27,28,35]. US was used in combination with CTA or MRI or as the only approach in patients included in screening programs [20,22,31,32,35].

Abdominal aortic initial diameter was reported in 13 studies and ranged between 19.9 and 50.9 mm. Only one study included aortas of less than 3 cm of diameter and studied aortic expansion rate through years [35]. Mean follow-up was estimated at 34.5 months (range 6–120 months). Aneurysm diameter at the end of surveillance was reported in seven studies and ranged between 20.3 and 55 mm. The annual growth rate was recorded in 12 studies. All data regarding diameters and growth rates are presented in Table 3. In three studies, aneurysm expansion was assessed providing volumetric data. Nakayama et al., Woloszko et al., and Tzirakis et al. provided data regarding aneurysm volume growth [30,31,36]. The estimated mean volume expansion was 8.14 cm^3^, 17 cm^3^ and 18.5 cm^3^, respectively [30,31,36]. Furthermore, Tzirakis et al.’s analysis reported a 6% annual aneurysm area expansion rate [36]. In two studies, the impact of infra-renal calcification (threshold at 50% of aneurysm circumferential) was studied, while two additional studies sub-analyzed that impact of thrombus on aneurysm expansion [26,30,32,38]. Furthermore, two studies evaluated the impact of ultrasmall superparamagnetic iron oxide (USPIO) and Sodium 18F-fluoride (18F-Na-F) enhancement on AAA evolution [29,33]. All data are provided in Table 3.

In 11 out of 23 studies, the initial diameter was characterized as a prognostic factor associated with aneurysm expansion. Only one study did not prove an association between the initial diameter and aneurysm growth rate [23]. The aforementioned study used finite element analysis. In an average follow-up time of 22 ± 13.6 months, initial aortic diameter was not found to be correlated with sac expansion (*p* = 0.19) while an association between peak wall stress and aneurysm expansion was recorded [23]. Regarding the presence of intra-luminal thrombus (ILT) and its impact on AAA expansion, 8 studies estimated its role; 7 studies concluded that presence of ILT affected positively aneurysm growth [16,25,27,31,32,36,38]. ILT distribution was evaluated by Behr et al. and concluded that the presence of circumferential ILT was associated to higher growth rate (2.09 mm/y) while in large aneurysms, ILT heterogeneity was detected [32]. In addition, George et al. associated the presence of inhomogeneous ILT to greater aneurysm growth [25]. In 3 studies, AAA wall calcification was evaluated in terms of growth rate; in 2 studies a negative association between atherosclerosis and aneurysm expansion was documented. In 3 studies, using finite elements, aneurysm volume was assessed and proved in one study, AAA volume better predicts aneurysm growth rate and correlates stronger with increasing estimated biomechanical rupture risk compared to diameter. Other factors, such as peak wall stress, AAA area and USPIO and 18F-Na-F, were assessed and evaluated regarding aneurysm expansion [29,33,34,37]. The commonest predictive factors are presented in Table 4.

## 4. Discussion

Current recommendations from Vascular Societies suggest AAA repair when the maximal aneurysm diameter achieves the 55 mm threshold [1,2,39]. For smaller diameter AAA, the European Society of Vascular Surgery suggests surveillance using US [1]. In the current endovascular era, the low mortality and rupture rates might permit the application of EVAR in smaller diameters, especially when considering the economic benefit of an elective procedure and the decreased psychological stress of a patient that needs to be re-evaluated yearly for an aneurysm that approaches diameter threshold [40,41,42,43]. Despite that rupture rates of small AAA appear to be low, aneurysm repair on smaller diameter seems technically feasible and safe with lower morbidity and mortality rates while the anatomical characteristics, as landing zones of small aneurysm are more “operator” friendly [43,44,45]. However, currently available data in the literature do not warrant firm conclusions regarding this state; no clear ascertainment and diagnostic criteria for small aneurysm rupture rate are provided [44]. The arising issue is to clarify the predictors of aggressive aneurysm growth in order not only to treat these patients before rupture but also to alternate the surveillance protocols in this specific group of AAA. For the moment, AAA diameter remains the gold standard as risk factor for rupture and indicator of repair [46].

CTA remains the gold standard of imaging in the pre-operative setting while US has established its role as the preferred imaging modality in screening, pre and post-operative surveillance [1]. Experimental studies using the application of modern imaging modalities as PET-CT and USPIO MRI suggest novel assessment methods in AAA evaluation where the increased nanoparticles enhancement associates to a more aggressive aneurysmal disease [27,33,34,46,47]. MRI may be used more frequently in the future due to its high sensitivity and specificity in tissue characteristics, no radiation and less medium contrast use. Additionally, the use of experimental computed modalities offers new diagnostic criteria in AAA risk assessment. Fluid structure interaction simulations using reconstructed CTAs have concluded that peak wall stress is associated with aneurysm expansion and can offer important information regarding the location of expansion or even, rupture [28,29,48]. Wall thickness and intraluminal thrombus presence were studied in the included analysis concluding in conflicting results [23,36]. Additional computed analyses regarding the aneurysm neck and iliac arteries alterations during AAA evolution have been providing scarce data [49,50].

Different parameters have been studied through years using the available imaging modalities. Despite that aneurysm diameter remains the most studied factor which associates to aneurysm progression and sets the indication for treatment [31,32,34,35,36,37,38,51,52], other visible AAA characteristics as ILT, calcification, and vascular anatomy have been studied and could be applied into daily clinical practice [46]. As the available data are limited, conclusions for the moment are controversial regarding the impact of ILT and atherosclerosis on AAA growth [20,25,26,27,30,31,48]. In general, atheromatosis of the aortic wall seems to offer a protective role in aneurysm expansion [20,30] while thrombus is associated to higher expansion rates in the majority of studies [25,27,30,31]. Nowadays, except imaging modalities, different biochemical factors, pharmaceutic and pathophysiologic pathways, as well as their effect on aneurysm expansion and risk of rupture are assessed and analyzed [53,54]. The association of biochemical markers and imaging features have been already performed in the current literature, offering promising results [21]. However, no association between imaging findings and blood circulating markers has been detected in the available studies [21]. Further analyses and novel approaches are needed to assess the role of the available imaging and biochemical entities on aneurysm progression [55,56,57].

In the future, technological evolution may assist the identification of individualized growth and rupture risk factors in AAA patients and may help discreet patients that may benefit from a sooner intervention. The clinical impact, regarding the risk of rupture and symptoms evolution, of these imaging markers has been presented in the current literature [16,17]. PET-CT has been used to provide such a relationship between the imaging findings and clinical evolution; AAAs that enhance a higher rate of nanoparticles are associated with a 3-fold higher risk of repair or ruptured, as well as a reduced chronological interval, from diagnosis to event [33]. Similar data are provided regarding the use of USPIO MRI as a higher enhancement rate was related to an elevated risk of rupture, repair or death [34]. The clinical impact of these imaging investigations is of high interest and permit the application of modern imaging techniques in a high risk population. The ideal approach may include the standard modalities to detect a group of patients at risk of rapid sac expansion and, further, the application of more sophisticated techniques on them to detect a more specific cohort that would benefit from an early repair.

### Limitations

Most of the included studies were retrospective, while no RCT was documented in the currently available literature. A high heterogeneity was detected in terms of study cohorts, initial aortic diameter and factors estimated and analyzed in its study. Furthermore, different imaging modalities, including US, CT, MRI, PET-CT and sophisticated computational models, were used to assess AAA characteristics.

## 5. Conclusions

AAA is a progressive disease with main treatment target of rupture prevention. Currently, aneurysm diameter is the most studied factor to be associated with aneurysm expansion and the main indication for intervention. In the future, appropriate software, including different anatomical characteristics, may identify aneurysm with rapid growth and higher rupture risk. Future perspectives, including computed mathematical models that will assess wall stress and elasticity and further flow characteristics, may offer valuable alternatives in AAA growth prediction.

## Figures and Tables

**Table 1:** P.I.C.O. (patient; intervention; comparison; outcome) model was used to define the clinical questions and clinically relevant evidence in the literature.

**P**	Patient, Population or Problem	Patients with AAA
**I**	Intervention, prognostic factor or exposure	Pre-operative surveillance with imaging modalities of patients with AAA
**C**	Comparison of intervention	AAA expansion rate during surveillance defined as the difference between the initial and latest available diameter devised by time (mm/year)
**O**	Outcome you would like to measure or achieve	Imaging findings associated to aneurysm growth
	What type of question are you asking?	Are there imaging factors that could predict AAA evolution?
		Are there imaging factors that affect positively or negatively aneurysm growth?
	Type of study you want to find	Cohort observational trials; prospective and retrospective reporting on AAA growth and predictive imaging findings

AAA: abdominal aortic aneurysm.

**Table 2:** Retrospective and 10 prospective. All patients underwent imaging with different modalities; the initial evaluation was followed by one or more studies to assess AAA (abdominal aortic aneurysm) expansion. Different modalities were applied including CTA, MRI, PET and US or a combination of them.

Author	Year	Journal	Study Period	Type	Imaging Modality
Wolf, et al. [16]	1994	JVS	1986–1992	Retrospective	CTA
Faggioli, et al. [17]	1994	Am J Surg	NA	Prospective	Angiography, CTA
Veldenz, et al. [18]	1994	Ann Vasc Surg	1988–1992	Retrospective	CTA
Kurvers, et al. [19]	2004	J Am Col Surg	1996–2002	Retrospective	CTA
Lindholt, et al. [20]	2008	Atherosclerosis	1994	Prospective	US
Speelman, et al. [21]	2010	EJVES	NA	Prospective	CTA
Badger, et al. [22]	2011	Vasc Med	2004–2006	Retrospective	US
Shang, et al. [23]	2013	JVS	NA	Retrospective	CTA
Farsad, et al. [24]	2015	J Cardiovasc Comput Tomogr	NA	Prospective	CTA
George, et al. [25]	2015	J Cardiovasc Comput Tomogr	2010–2011	Retrospective	CTA
Hendy, et al. [26]	2015	Atheroscl	2003–2013	Prospective	CTA
Huang, et al. [27]	2016	Mol Imaging	NA	Prospective	PET-CT, CTA
Joly, et al. [28]	2016	Comput Biol Med	2006–2013	Retrospective	CTA, MRI
Lindquist, et al. [29]	2016	JVS	2009–2013	Retrospective	CTA
Nakayama, et al. [30]	2016	Circ J	2003–2011	Retrospective	CTA
Woloszko, et al. [31]	2016	Med Sci Monit	2005–2010	Prospective	US, CTA
Behr, et al. [32]	2017	J Cardiovasc Surg	2014–2015	Retrospective	MRI, US
Forsythe, et al. [33]	2017	JACC	NA	Prospective	PET-CT
MARS investigators [34]	2017	Circ	2012–2014	Prospective	MRI
Nyronning, et al. [35]	2019	Scand Cardiovasc J	1994–2005	Prospective	US
Tzirakis, et al. [36]	2019	Ann Vasc Surg	NA	Retrospective	CTA
Hirata, et al. [37]	2020	J Comput Assist Tomogr	2010–2016	Retrospective	CTA
Zhu, et al. [38]	2020	Radiology	2004–2018	Retrospective	CTA, MRI, PET CT

CTA: computed tomography angiography; US: ultrasound; MRI: magnetic resonance imaging; NA: not applicable; PET-CT: positron emission tomography-computed angiography.

**Table 3:** Abdominal aortic initial diameter was reported in 13 studies and ranged between 19.9 and 50.9 mm.

Authors	AAA Diameter Threshold for Inclusion	Initial AAA Diameter	Follow-Up (Months)	AAA Diameter at Follow-Up	AAA Growth Rate in mm/year
Wolf, et al. [16]	>30 mm	44 ± 6 mm	22 ± 12	NA	2.5 ± 2.4
Faggioli, et al. [17]	<50 mm	NA	NA	NA	NA
Veldenz, et al. [18]	<50 mm	NA	15	NA	NA
Kurvers, et al. [19]	NA	50 ± 9 mm	42	NA	3.6 ± 2.4
Lindholt, et al. [20]	NA	32 mm	6.15 ± 3.61	NA	2.45
Speelman, et al. [21]	NA	NA	12	NA	NA
Badger, et al. [22]	46 mm	39 mm	NA	NA	0.75 for AAA <35 mm & 4.32 for AAA >50 mm
Shang, et al. [23]	NA	45.8 ± 7.7 mm	22.0 ± 13.6	50.6 ± 9.0 mm	2.8 ± 1.7
Farsad, et al. [24]	<5 cm	NA	NA	NA	NA
George, et al. [25]	NA	NA	26	NA	NA
Hendy, et al. [26]	NA	NA	16	NA	1.6 vs. 1.8 in AAA with > or < than 50% of wall calcification, respectively
Huang, et al. [27]	NA	41 ± 5.4 mm	NA	NA	NA
Joly, et al. [28]	<55 mm	NA	96	NA	NA
Lindquist, et al. [29]	<50 mm	52 mm	12	55 mm	3.1
Nakayama, et al. [30]	<55 mm	44.7 ± 14.6 mm	19	52.9 ± 2.9 mm	NA
Woloszko, et al. [31]	NA	39 mm	24	43 mm	NA
Behr, et al. [32]	>30 mm	31.9 mm	67	42.3 mm	1.95; 2.04 in case of circumferential thrombus
Forsythe, et al. [33]	<50 mm	NA	16.7± 6.4	NA	2.20
MARS investigators [34]	NA	49.6 ± 7.7 mm	33 ± 9.2	NA	2.8 ± 2.4
Nyronning, et al. [35]	<40 mm	19.9 mm	120	20.3 mm	3.1 in 2 years of FUP
Tzirakis, et al. [36]	>40 mm	NA	NA	NA	3.35
Hirata, et al. [37]	NA	42.8 ± 6.7 mm	NA	NA	3.0 ± 2.3
Zhu, et al. [38]	32–56 mm	38 mm	39.6 ± 30	44 mm	1.5; 2.0 in AAA with intra-luminal thrombus

Mean follow-up was estimated at 34.5 months (range 6–120 months). Aneurysm diameter at the end of surveillance was reported in 7 studies and was ranging between 20.3 and 55 mm. The annual growth rate was recorded in 12 studies. AAA: abdominal aortic aneurysm; NA: not applicable; FUP: follow-up.

**Table 4:** Out of 23 studies, the initial diameter was characterized as a prognostic factor associated to aneurysm expansion.

Author	Number of Imaging Predictive Factors Per Study	Initial AAA Diameter	Presence of Intra-Luminal Thrombus	Type of Thrombus Associated to Expansion	Presence of Aortic Wall Calcification	AAA Volume
Wolf, et al. [16]	1		Positive			
Faggioli, et al. [17]	1					
Veldenz, et al. [18]	1					
Kurvers, et al. [19]	1	Positive				
Lindholt, et al. [20]	1				Negative	
Speelman, et al. [21]	1					
Badger, et al. [22]	2	Positive				
Shang, et al. [23]	2	No associated				
Farsad, et al. [24]						
George, et al. [25]	2	Positive	Positive	Inhomogeneous		
Hendy, et al. [26]	2				Positive	
Huang, et al. [27]	2		Positive			
Joly, et al. [28]	1					
Lindquist, et al. [29]	1					Positive
Nakayama, et al. [30]	2	Positive			Negative	
Woloszko, et al. [31]	3	Positive	Positive			
Behr, et al. [32]	1	Positive	Positive	Inhomogeneous Circumferential		
Forsythe, et al. [33]	1					
MARS investigators [34]	3	Positive				
Nyronning, et al. [35]	2	Positive				
Tzirakis, et al. [36]	3	Positive	Positive			Positive
Hirata, et al. [37]	2	Positive	Not associated			
Zhu, et al. [38]	2	Positive	Positive			

Regarding the presence of intra-luminal thrombus, seven studies concluded that presence of thrombus affected positively aneurysm growth. Aneurysm wall calcification was documented to have a negative association to aneurysm expansion in two out of three studies. AAA: abdominal aortic aneurysm.

## Data Availability

No new data were created or analyzed in this study. Data sharing is not applicable to this article.

## Supplementary Materials

The following are available online at https://www.mdpi.com/article/10.3390/jcm10091917/s1, Table S1: The quality of observational studies was assessed using the Newcastle-Ottawa Scale (NOS).
