# Supplementary material for: Imaging Predictive Factors of Abdominal Aortic Aneurysm Growth
Source: J Clin Med. 2021 Apr 28;10(9):1917. doi: 10.3390/jcm10091917 (PMC8124923; doi:10.3390/jcm10091917)
Supplement: Supplementary file 1 [file jcm-10-01917-s001.zip › jcm-1185170-supplementary.pdf]

Table S1. The quality of observational studies was assessed using the Newcastle-Ottawa Scale (NOS)

**Study**	**Selection**	**Comparability**	**Outcome**
Wolf, *et al.*[16]	****	*	**
Faggioli, *et al.*[17]	****	*	*
Veldenz, *et al.*[18]	****	*	***
Kurvers, *et al.*[19]	****	*	* **
Lindholt, *et al.*[20]	****	**	*
Speelman, *et al.*[21]	****	**	***
Badger, *et al.*[22]	****	*	***
Shang, *et al.*[23]	**	*	*
Farsad, *et al.*[24]	*	*	*
George, *et al.*[25]	****	**	***
Hendy, *et al.*[26]	****	**	***
Huang, *et al.*[27]	***	*	*
Joly, *et al.*[28]	**	*	***
Lindquist, *et al.*[29]	****	**	***
Nakayama, *et al.*[30]	***	**	***
Woloszko, *et al.*[31]	****	**	***
Behr, *et al.*[32]	****	**	***
Forsythe, *et al.*[33]	***	**	***
MARS investigators[34]	****	**	***
Nyronning, *et al.*[35]	****	*	***
Tzirakis, *et al.*[36]	**	*	*
Hirata, *et al.*[37]	***	**	*
Zhu, *et al.*[38]	***	**	***
